# Artificial intelligence-based language learning: illuminating the impact on speaking skills and self-regulation in Chinese EFL context

**DOI:** 10.3389/fpsyg.2023.1255594

**Published:** 2023-11-02

**Authors:** Hongliang Qiao, Aruna Zhao

**Affiliations:** ^1^School of Foreign Languages, Northeast Petroleum University, Daqing, China; ^2^Department of Foreign Language, Baotou Teachers’ College, Inner Mongolia University of Science and Technology, Baotou, China

**Keywords:** artificial intelligence, AI-based instruction, speaking skills, self-regulation, Duolingo, EFL

## Abstract

**Introduction:**

This study investigated the effectiveness of artificial intelligence-based instruction in improving second language (L2) speaking skills and speaking self-regulation in a natural setting. The research was conducted with 93 Chinese English as a foreign language (EFL) students, randomly assigned to either an experimental group receiving AI-based instruction or a control group receiving traditional instruction.

**Methods:**

The AI-based instruction leveraged the Duolingo application, incorporating natural language processing technology, interactive exercises, personalized feedback, and speech recognition technology. Pre- and post-tests were conducted to assess L2 speaking skills and self-regulation abilities.

**Results:**

The results of the study demonstrated that the experimental group, which received AI-based instruction, exhibited significantly greater improvement in L2 speaking skills compared to the control group. Moreover, participants in the experimental group reported higher levels of self-regulation.

**Discussion:**

These findings suggest that AI-based instruction effectively enhances L2 speaking skills and fosters self-regulatory processes among language learners, highlighting the potential of AI technology to optimize language learning experiences and promote learners’ autonomy and metacognitive strategies in the speaking domain. However, further research is needed to explore the long-term effects and specific mechanisms underlying these observed improvements.

## 1. Introduction

In recent years, the integration of information technology into education has revolutionized instructional methodologies, with portable computers becoming ubiquitous tools in educational organizations (Ruiz-Mercader et al., 2006; Gikas and Grant, 2013; Shatri, 2020; Sheikh et al., 2021; Akhmedov, 2022; Fathi et al., 2023; Garcia and Garcia, 2023; Liu et al., 2023). This technological surge has empowered educational institutions to enrich their curricula by incorporating virtual learning environments for instructional tasks (Hamuddin, 2018; Yamamoto et al., 2018). Among the array of technological advancements, artificial intelligence (AI) has emerged as a potent force in the realm of education, particularly in the domain of spoken communication, which has witnessed remarkable progress (Pokulevska, 2018). The integration of AI is driven by the aim to facilitate language learning within virtual environments, emancipating learners from the constraints of time and physical classroom contexts, thus enabling seamless access to course materials and fostering communication with teachers and peers (Hamuddin, 2018; Ahmad et al., 2021; Gardner et al., 2021).

Furthermore, AI has proven to be a transformative tool in kindling students’ enthusiasm and facilitating interactive language learning activities, indispensable in the contemporary educational landscape (Wekke et al., 2017). Notably, the field of English as a Foreign Language (EFL) has witnessed the positive influence of AI in recent years (Hamuddin, 2018; Junaidi, 2020). Through automatic speech recognition technology, AI systems exhibit an ability akin to human comprehension, detecting and comprehending learners’ speech. This capacity is particularly valuable in environments where native English speakers may not be readily available, effectively bolstering learners’ speaking skills (Junaidi, 2020; Zou et al., 2020; Soleimani et al., 2022). It is pertinent to acknowledge that while the application of AI in this context is promising, it remains in its nascent stages, with substantial strides in automatic speech recognition technology materializing only in the early 2010s (Johnson and Valente, 2009).

The efficacy of AI in augmenting English language learning performance has been subject to extensive investigation (Aljohani, 2021; Sun et al., 2021; Zhang, 2022; Huang et al., 2023). Huang et al. (2023) conducted a comparative study assessing the learning performance and engagement of learners in an AI-driven class, offering personalized video selection options, juxtaposed with a non-AI class devoid of such choices. The results unveiled superior learning outcomes and heightened engagement levels among learners in the AI-driven class.

Moreover, a multitude of studies have delved into the impact of AI on the development of speaking skills among English language learners (Hill et al., 2015; Junaidi, 2020; Maknun, 2020; Divekar et al., 2022; Kang, 2022; Suciati et al., 2022; Rustamova and Rakhmatullaeva, 2023). For instance, Hill et al. (2015) scrutinized human-human interactions in contrast to AI-human interactions, revealing that learners exhibited prolonged engagement in interactions with AI compared to their interactions with peers. Similarly, Kang (2022) compared learner-AI interactions with learner-native speaker interactions, uncovering the pivotal role of AI in enhancing learners’ speaking skills. Junaidi (2020) further substantiated these findings, indicating that AI-supported instruction positively impacted learners’ overall speaking performance, including aspects of fluency, grammatical accuracy, lexicon, and pronunciation.

Self-regulated learning (SRL) stands as a foundational concept in the educational landscape and has garnered considerable attention, particularly within the domain of language learning. Zimmerman (1989) aptly defines SRL as the active process through which learners proactively manage and oversee their cognitive, metacognitive, motivational, and emotional dimensions in pursuit of their learning objectives. This multifaceted construct encompasses a wide array of strategies and processes, including goal establishment, self-monitoring, strategic planning, metacognitive awareness, and the regulation of motivation (Zimmerman, 2002). SRL empowers learners to take control of their learning experiences, adapt to shifting demands, and optimize their learning outcomes (Pintrich, 2000). It unfolds as a cyclical journey of forethought, performance, and self-reflection, with individuals setting goals, applying strategies, and evaluating their progress, all of which significantly influence their academic achievements (Zimmerman, 2002).

In the realm of language learning, SRL plays a pivotal role in shaping learners’ linguistic proficiency and autonomy. It empowers them to actively engage with linguistic content, adeptly manage their learning resources, and navigate the intricacies of language tasks (Teng and Zhang, 2016). Scholars have underscored the critical importance of investigating SRL in language learning contexts as it holds the potential to enhance pedagogical practices and foster learners’ self-directedness (Oxford, 2016; Bowen and Thomas, 2022). Understanding how learners regulate their language learning processes, establish goals, and employ strategies is paramount for educators striving to design effective language instruction and cultivate autonomous language learners.

In this study, the operational definition of SRL is anchored in Seker’s (2016) model, which draws inspiration from well-established models of SRL in the realm of second language acquisition, primarily building upon Boekaerts (1997) model and Oxford’s (1990) L2 learning strategy inventory. The examination of self-regulated learning takes on pivotal significance within this study, as it serves as a linchpin for comprehending the intricate dynamics that unfold when AI-based instruction intersects with learners’ active involvement, motivation, and cognitive strategies during their language learning journey (Chang, 2005). This comprehensive investigation not only sheds light on how AI impacts these critical facets but also offers a deeper insight into the mechanisms that underpin effective language acquisition. Consequently, the findings gleaned from this study are poised to offer invaluable guidance for refining and enhancing pedagogical approaches tailored to the unique needs and preferences of language learners in AI-integrated educational settings.

Despite previous research delving into the influence of AI on the speaking skills of English language learners, a notable gap persists in comprehending the impact of AI on the speaking skills and self-regulation of EFL learners. Consequently, further investigation is imperative to elucidate the role of AI in enhancing both the speaking skills and self-regulation of EFL learners. This study, therefore, endeavors to bridge this research gap, aiming to meticulously examine the effects of AI on the speaking skills and self-regulation of Chinese EFL learners. In this Randomized Controlled Trial (RCT), we employed two dependent variables: speaking skills, operationalized as a composite of skills encompassing fluency, vocabulary, accuracy, and pronunciation, and self-regulation. Additionally, we considered speaking anxiety and global English proficiency as control variables, providing a comprehensive understanding of the multifaceted impacts of AI in the educational landscape. Speaking anxiety, in the context of this study, refers to the emotional and psychological discomfort or apprehension experienced by language learners when engaging in L2 spoken interactions. It encompasses feelings of nervousness, fear, or unease related to speaking tasks or situations in the L2 learning environment (Ozdemir and Papi, 2022). The ensuing findings hold substantial practical and pedagogical implications for the EFL context, furnishing valuable insights for educators and practitioners alike.

## 2. Literature review

### 2.1. Theoretical framework

The collaborative speaking exercises employed in this study align with the principles of social constructivism, as proposed by Vygotsky (1984), within both participant groups. Vygotsky (1984) argued that interactions with individuals who possess greater skills and intellect enable learners to progressively internalize knowledge and develop higher levels of autonomous consciousness. According to Vygotsky (1986), “every function in the child’s cultural development appears twice: first, on the social level, and later, on the individual level; first, between people (inter-psychological), and then inside the child (intra-psychological)” (p. 57). The concept of the zone of proximal development (ZPD), at the core of Vygotsky’s social constructivist theory, refers to the disparity between learners’ current level of problem-solving ability and their potential for improvement when engaged in collaborative problem-solving activities with more competent peers. In the learning environment, learners can reach their ZPD by actively participating in activities and seeking assistance from others. By attaining their ZPD, learners are able to independently regulate their learning tasks and exhibit greater autonomy within the learning context.

Consistent with the findings of Hsu et al. (2023) and Kim (2008), collaborative learning in a group enables students to support each other in reaching their ZPD across various language learning abilities. This is achieved by assuming roles as more proficient or less proficient learners depending on the language learning tasks and activities. By collaborating in pairs or groups to accomplish diverse language learning tasks, students engage in co-constructing language learning competencies and reaching their ZPD. In the present study, both the experimental and control groups adhered to Vygotsky’s social constructivist theory of learning, involving interactive speaking activities with peers. In the experimental group, students interacted with AI to access their ZPD, while in the control group, students achieved their ZPD through collaborative speaking activities with more proficient peers.

### 2.2. Artificial intelligence

AI has emerged as an effective pedagogical approach in language learning and instruction, offering language learners various opportunities to enhance their language learning performance (Zhang and Zou, 2020; Sun et al., 2021; Zhang, 2022) and foster positive perceptions and attitudes toward AI (Xia et al., 2022). Aldosari (2020) defines AI as a programmed system that simulates and produces intelligent applications for computers and smartphones, capable of performing a wide range of tasks with human assistance. Luckin et al. (2016) argue that AI can provide support in teaching, group learning, and virtual reality within language learning contexts.

Bibauw et al. (2019) suggest that AI-supported teaching, such as chatbots, facilitates communication between learners and provides both input and output. Chatbots promote authentic and meaningful social interactions (Clark, 2018) with various modalities, including text, audio, and visual elements, while delivering prominent and easily comprehensible feedback (Bao, 2019). According to Akerkar (2014) and Ginsberg (2012), AI possesses the capability to make informed and intelligent decisions comparable to those made by humans. Kim (2018) highlights the precise evaluation and enhanced features of AI applications, making them an effective means to improve oral skills, particularly in listening and speaking.

Several studies have been conducted addressing the impact of AI on different language learning skills in English as a second and foreign language contexts, including writing (Fitria, 2023), reading (Liu, 2021), listening (Suryana et al., 2020), and speaking (Maknun, 2020; Divekar et al., 2022; Rustamova and Rakhmatullaeva, 2023). With regard to speaking skills, Suciati et al. (2022), for example, qualitatively examined the impact of an AI-based program, namely Cake on learners’ language learning and their speaking abilities. Collecting the required data *via* interview, observation, and documentation, the findings indicated that AI-based instruction had a significant impact on the learners’ language learning in general and their speaking abilities in particular. Some features of AI-based instruction, such as the user-friendliness of the program, its availability in different places and at different times, its wide repertoire of speaking topics, and the ability to evaluate learners’ language learning, were believed to be the reason behind the learners’ successful performance in language learning and speaking performance.

In a quasi-experimental study conducted by Maknun (2020), the impact of an AI application called Orai on EFL students’ speaking performance was examined. The experimental group utilized Orai for their communicative speaking activities, while the control group did not incorporate Orai into their group-based speaking interactions. The findings demonstrated that the experimental students exhibited higher speaking performance compared to the control students after the intervention, underscoring the significant role of AI-based instruction in enhancing EFL students’ speaking abilities.

Similarly, Safadi et al. (2022) investigated the influence of AI-based instruction on the speaking skills of female English language learners using a quasi-experimental research design. In this study, the experimental group engaged in interaction with AI to develop their speaking performance, while the control group’s speaking performance was enhanced through interactive speaking activities with peers. Speaking skills tests were administered to collect the necessary data, and the findings revealed that the experimental learners surpassed the control learners in speaking performance, thereby confirming the substantial effects of AI-based instruction in fostering the speaking performance of female English language learners.

In a similar vein, Junaidi (2020) investigated the role of AI-supported instruction using Lyra application in improving EFL learners’ speaking skills *via* an experimental, control group research design. The learners using their mobile phones to communicate with AI Lyra during class time in order to develop their speaking skills. The control group, however, did not utilize the AI Lyra during their interactive speaking activities. The learners in both groups mainly focused on the sub-scales of speaking performance, namely fluency, grammatical accuracy, lexicon, and pronunciation. The results demonstrated that the AI-supported instruction outperformed its non-AI counterpart in developing the speaking subcomponents of the EFL learners. Makhlouf (2021) also examined the effectiveness of AI instruction on EFL learners’ speaking performance in general and fluency and accuracy, as the subcomponents of speaking performance, in particular. The study adopted a one-group pre-test, and post-test research design and gathered the required data through some developed speaking skills tests. The study examined whether the learners’ interactions with AI using their mobile devices during class time affected their speaking performance. The findings showed the learners’ improvements in speaking performance in general and fluency and accuracy in particular.

Kang (2022) examined the differences between AI-and native speaker-supported instruction on speaking skills and affective factors of second language learners in a college in the United States. The findings indicated that both AI-supported instruction and native speaker instruction significantly developed the learners’ speaking skills. However, the learners who had interaction with AI outperformed those who had interaction with a native speaker in enhancing their speaking performance in general and speaking skills, including accuracy, fluency, and coherence. The findings further revealed that the learners with a low proficiency level benefited more from interaction with AI in comparison with the learners with a high proficiency level. On the other hand, the learners with a high proficiency level benefited more from interaction with a native speaker. Finally, the AI learners held positive perceptions toward AI-based interaction, while native-speaker interaction did not positively change the learners’ perceptions toward native-speaker-based interaction.

In addition, El Shazly (2021) investigated the impact of AI on the speaking performance and anxiety of EFL learners *via* a quasi-experimental, pretest–posttest design. Collecting the required data *via* an anxiety test and IELTS speaking skill test, the results demonstrated that AI significantly improved the learners’ speaking performance, however, AI did not diminish the learners’ anxiety level. Hill et al. (2015) compared human-human interaction with human-AI interaction using the chatbot Cleverbot. The two types of interactions were explored by considering the number of words that occurred in each message and conversation, the number of messages in each conversation, and the uniqueness of the words that occurred in each utterance. The findings showed that human-AI interaction took a longer period of time in comparison with human-human interaction but contained shorter messages. The findings also demonstrated that human-human interaction contained rich vocabulary elements in comparison with human-AI interaction which lacked the richness of vocabulary in conversations.

In Yang et al.’s (2022) study, they introduced an innovative task-based voice chatbot named “Ellie” as an English conversation partner. The results indicated that students enthusiastically engaged with Ellie, and the high task success rates demonstrated the appropriateness of the chatbot’s task design and operational intents. While the research emphasized the positive potential of chatbots in EFL environments, it also acknowledged specific limitations that warrant attention, offering valuable insights for the future integration of AI chatbots in language classrooms.

Hsu et al. (2021) conducted an investigation into the influence of the Amazon Echo Show, a widely used Intelligent Personal Assistant (IPA), on the listening and speaking skills of L2 learners. Their controlled experiment encompassed two groups, revealing notable enhancements in speaking proficiency among learners who interacted with the IPA. Furthermore, learners reported that IPAs provided increased opportunities for oral interactions and alleviated speaking anxieties. Divekar et al. (2022) merged AI and extended reality (XR) to create lifelike conversational interactions for the acquisition of foreign languages. Their seven-week evaluation, involving university students learning Chinese as a foreign language, demonstrated significant enhancements in vocabulary, comprehension, and conversational skills, underscoring the effectiveness of AI in language learning.

Also, Yan (2023) adopted a qualitative approach to explore the impact of ChatGPT in L2 writing classrooms. The findings indicated that ChatGPT had the potential to enrich L2 writing pedagogy by introducing developmental features in learning activities and facilitating efficient writing. This pioneering endeavor underscored the need for further research into ChatGPT’s applicability in L2 learning and the formulation of corresponding pedagogical adaptations. Jia et al. (2022) devised an AI system designed to enable authentic and ubiquitous language acquisition. Their study, involving 20 participants, employed a combination of research methods to assess the system’s usability and validity. The findings affirmed the efficacy of the AI system and yielded insights for potential enhancements. This research contributes to the integration of AI in language instruction and adheres to mobile learning principles, emphasizing the significance of authentic learning environments.

With regard to Duolingo application, Li and Bonk (2023) conducted a study on online language learners using Duolingo outside of formal classrooms. They found that learners employed various resources and self-monitored their learning process, relying on Duolingo’s features. Intrinsic motivations, such as cultural interest and travel, drove learners more than certificates or grades. Kessler (2023) addressed limitations in mobile-assisted language learning (MALL) applications by integrating reflective e-journal activities with Duolingo. The study, grounded in metacognition theory, revealed that the journals enhanced students’ metacognitive awareness in various domains, with learners finding the activity beneficial and enjoyable. Also, Shortt et al. (2023) reviewed Duolingo’s gamified MALL application, highlighting its popularity and gamification elements. They found that research focused on app design, quantitative methods, and non-probability sampling, emphasizing tool creation over learning process and outcomes. The review identified preferences for performance-based research questions, English language, and the United States as the main research context, revealing research gaps with implications for MALL and gamification practitioners and researchers.

The reviewed studies collectively underscore the transformative potential of AI in language learning, particularly in honing speaking skills. Each of these interventions has demonstrated significant enhancements in learners’ speaking performance, providing evidence of the substantial impact AI can have in language education. However, despite the promising strides made in understanding the impact of AI on language learning, a notable gap persists in comprehending its effects in specific contexts, such as the Chinese EFL environment examined in this study. While existing research provides valuable insights, there remains a need for a more nuanced exploration of AI’s role in enhancing speaking skills within this specific demographic. This study aims to address this gap by conducting a rigorous investigation into the impact of AI-powered language learning on speaking skills and self-regulation among Chinese EFL learners, contributing to the broader discourse on the integration of AI in language education.

### 2.3. Purpose of the current study

The existing literature has demonstrated the positive influence of AI on the language learning performance of English language learners (Wang, 2022). Additionally, several studies have highlighted the significant role of AI in improving English language learners’ speaking performance (Hill et al., 2015; Junaidi, 2020; Maknun, 2020; Kang, 2022; Suciati et al., 2022). However, there is a research gap regarding the investigation of AI’s impact on learners’ speaking skills and self-regulation, particularly in the EFL context. To address this gap, the present study aimed to examine the effects of AI on the speaking skills and self-regulation of EFL learners. To achieve this goal, a randomized controlled trial (Benson and Hartz, 2000) was conducted. Participants were randomly assigned to either the treatment group or the control group. Both groups received instruction across a span of 13 weeks, with each week comprising one session. In the treatment group, participants received AI-based instruction, while the control group received instruction without AI. To assess the effectiveness of the AI-based instruction on speaking skills, four measures including fluency, vocabulary, accuracy, and pronunciation were used. Additionally, two control variables were measured to account for the possible influence of students’ language proficiency and speaking anxiety on the dependent variable. The first control variable was global English proficiency, which was measured using College English Test (CET). The second control variable was speaking anxiety, which was included due to its potential impact on speaking performance.

To further explore the impact of the AI-based instruction, the interaction term of the course and pretest score was included as an additional predictor variable. This allowed us to assess the differential effects of the intervention for EFL students with low versus high pretest scores on the dependent variable. Based on the literature, we expected that the AI-based speaking instruction would lead to improvements in students’ speaking skills and self-regulation, as reflected by the four measures used in this study.

## 3. Method

### 3.1. Participants

The present study involved 93 intermediate-level EFL students in Mainland China. The sample included both male (*n* = 41) and female (*n* = 52) participants. These students were enrolled in one of four conversation courses offered by five different institutes that offered the AI-based speaking instruction. The institutes participating in this study were a mix of English language institutes and university programs, each recognized for their commitment to providing quality language education. Although there were no significant differences in the demographic characteristics or English language proficiency levels of students across the institutes, it is worth noting that they offered a variety of supplementary resources and support services to facilitate language learning. These included language labs, conversation clubs, and access to digital learning platforms, all of which contributed to the overall learning experience.

The demographics of the participants were as follows: the mean age was 21.36 years old (SD = 2.86), with a range of 19 to 26 years. The majority of participants reported Chinese as their first language (97%), while the remaining 3% spoke other languages at home.

To evaluate the effectiveness of the AI-based speaking instruction, a randomized controlled trial with repeated measures was conducted (Deaton and Cartwright, 2018). Prior to the study, all participants provided informed written consent. Pretest measurements were collected before the intervention started, embedded within the first two course sessions, and posttest measurements were collected in the last course session. At each institute, one control group participated in a traditional speaking course, while the intervention group received the AI-based speaking instruction. Both courses were offered simultaneously, with the AI-based instruction being delivered by a team of researchers and two trained English teachers who collaborated with the present researcher. The AI-based speaking instruction was identical for all groups.

The two English teachers involved in delivering AI-based instruction possessed extensive experience in teaching English as a foreign language, with a combined teaching experience of over 20 years. Both instructors held advanced degrees in TESOL (Teaching English to Speakers of Other Languages) and were well-versed in the principles of language pedagogy and technology-enhanced language learning. Moreover, they underwent specialized training in utilizing AI-based language learning platforms and were proficient in managing and supporting learners within this context.

To achieve randomization, the two courses, namely the AI-based course and the conventional course, were presented as part of a course-tandem known as the ‘English Speaking Course.’ This course-tandem approach involved students enrolling in both the AI-based and conventional courses simultaneously, allowing them to experience both instructional methods. To ensure a fair and unbiased distribution of students between the control and experimental groups across all participating institutes, a blocked randomization technique, aided by computer-generated random numbers, was applied. This process was designed to establish an equitable representation of participants in both instructional groups, thus minimizing potential bias in group assignments. In total, 47 students were randomly assigned to the AI-based instruction (age: *M* = 20.2, SD = 1.8; 45.5% female), and 46 students were assigned to the traditional speaking course (age: *M* = 20.1, SD = 1.7; 37.5% female). After the study, all students were invited to participate in the respective other course, allowing for a crossover design to assess the persistence of the AI-based instruction’s effect on speaking skills.

### 3.2. Instruments

#### 3.2.1. Speaking skills

To assess the effectiveness of the AI-based speaking instruction on the students’ speaking skills, four measures of speaking skill components were used: fluency, vocabulary, accuracy, and pronunciation. The speaking abilities of Chinese EFL learners were evaluated using the IELTS speaking skill examination. This assessment encompassed four equally weighted components, namely fluency and coherence, vocabulary, grammatical range and accuracy, and pronunciation. The learners’ performance in each area was evaluated based on the topics provided in the IELTS speaking test. The IELTS Speaking Band Descriptors were employed to assign scores ranging from 1 to 9 to each learner in each speaking skill category. These individual scores were then aggregated and divided by four to determine the overall speaking score for each student. To ensure consistency, the speaking skills of the learners were evaluated by two proficient assessors, consisting of the researcher and another experienced instructor specialized in teaching EFL speaking. The inter-rater reliability, assessed using the Cohen’s kappa coefficient, yielded a satisfactory value of 0.87.

#### 3.2.2. Self-regulated learning

To assess the participants’ L2 self-regulation, we employed the Self-Regulated Language Learning Questionnaire (SRLLQ), originally developed and rigorously validated by Seker (2016). This Likert-scale instrument offers five response options and serves as a tool to gage learners’ self-reported engagement in Self-Regulated Learning (SRL), drawing on well-established models within the field. The SRLLQ consists of a comprehensive set of 30 items, thoughtfully distributed across five distinct subscales, namely internal motivation (*n* = 5), external motivation (*n* = 4), cognitive strategies (*n* = 7), metacognitive strategies (*n* = 10), and evaluation (*n* = 4). Notably, great care was taken in crafting the questionnaire items, ensuring that they employ straightforward language and phrasing, making them easily accessible to students without the need for translation. The SRLLQ demonstrated excellent internal consistency reliability, with Cronbach’s alpha values of 0.85 at the pretest and 0.79 at the posttest, reaffirming its robustness within our study’s sample.

#### 3.2.3. Control variables

To ensure the accuracy and reliability of the results, control variables were included in the study (Cohen et al., 2003). Firstly, the global English proficiency of participants was assessed using the College English Test 3 (CET-3). CET-3 is a well-established standardized English proficiency examination widely recognized in China. It is a part of the reputable College English Test (CET) series, which is meticulously organized and overseen by the National College English Testing Committee, an authoritative body in English language assessment (Wang, 1999). CET-3 is specifically designed for college students who have completed a three-year English study as part of their undergraduate programs. This comprehensive test rigorously evaluates students’ proficiency in key language skills including listening, reading, and writing. It encompasses various critical aspects of English language competence such as vocabulary, grammar, comprehension, and written expression.

Importantly, CET-3 scores hold significant recognition and are extensively utilized by Chinese universities as a pivotal criterion for assessing students’ English proficiency levels. These scores play a crucial role in evaluating eligibility for graduation and various academic pursuits.

It is worth noting that the widespread use and acceptance of CET-3 scores in Chinese universities have been affirmed by numerous studies (Yan and Huizhong, 2006; Li, 2009). Additionally, the test’s validity and reliability have been rigorously examined and documented in a range of academic publications (Zheng and Cheng, 2008). The endorsement of CET-3 by reputable institutions further attests to its credibility and effectiveness in evaluating English language proficiency among Chinese students (Xu and Liu, 2018).

Furthermore, to account for the potential influence of anxiety on speaking performance, we incorporated a measurement of speaking anxiety. We evaluated participants’ anxiety levels while engaging in L2 speaking activities using a meticulously validated 19-item questionnaire developed by Ozdemir and Papi in 2022. This scale, stemming from the foundational Foreign Language Classroom Anxiety (FLCA) Scale originally conceptualized by Horwitz et al. (1986), was thoughtfully adapted to specifically target anxiety within speaking contexts. Participants were asked to rate their responses to each item on a 6-point Likert scale, allowing them to express their agreement or disagreement, ranging from ‘strongly agree’ to ‘strongly disagree.’

Finally, the pre-test scores were measured to account for any differences in the participants’ initial speaking skills before the intervention began. Including these control variables increased the precision of the regression coefficients and reduced any potential bias that may have been caused by differences between the two groups at the beginning of the study. The use of these control variables ensured that any improvements in the participants’ speaking skills and self-regulation could be attributed to the AI-based speaking instruction, rather than other factors such as overall English proficiency or anxiety levels.

#### 3.2.4. AI-based language learning application: Duolingo

Duolingo stands at the forefront of language learning applications, acclaimed for its pioneering approach to language acquisition. Since its inception in 2011, Duolingo has evolved into a globally recognized and widely adopted language learning platform, earning its place as an AI-powered language learning tool of choice.

A cornerstone of Duolingo’s language learning curriculum is its immersive speaking exercises. These exercises immerse learners in spoken interactions with the application, requiring them to respond to prompts and questions in their target language. These interactions play a pivotal role in honing speaking fluency as they compel learners to articulate their thoughts and ideas verbally.

A standout feature within Duolingo’s speaking component is its real-time feedback mechanism. As learners respond to prompts, an AI-powered chatbot diligently evaluates various facets of their spoken language, encompassing pronunciation, fluency, vocabulary usage, and grammatical accuracy. This instantaneous feedback mechanism is made possible by the application of machine learning algorithms, meticulously analyzing learner performance data. Consequently, learners receive personalized feedback tailored to their unique linguistic requirements, enabling them to effect immediate refinements and improvements in their speaking skills (Kessler, 2023). Also, Duolingo places a strong emphasis on motivation through gamification elements seamlessly integrated into its platform. Learners embark on a journey where they earn points, conquer challenges, and unlock new levels as they progress, nurturing a sense of achievement and fostering consistent practice. The application further empowers learners to track their language learning odyssey, offering insights into their overall progress and areas that may warrant additional focus (Li and Bonk, 2023).

In addition to individual speaking exercises, Duolingo offers interactive group activities and discussions that replicate real-world conversational scenarios (Shortt et al., 2023). These features immerse learners in dynamic and naturalistic contexts, refining their ability to comprehend and respond to spoken language in real time. Engaging in dialogs and discussions enriches their speaking skills. The infusion of AI technology into Duolingo represents a groundbreaking shift in language acquisition. It provides learners with uninterrupted, interactive, and personalized practice opportunities. Through its speaking exercises and real-time feedback, Duolingo propels learners toward speaking fluency by encouraging them to express their thoughts and ideas in the target language, simultaneously refining their pronunciation and language production abilities.

In our study, we harnessed the formidable capabilities of the Duolingo application, with its AI-driven speaking component, to scrutinize its precise impact on speaking skills, prominently fluency, within the context of Chinese EFL learners. By harnessing the rich feature set of Duolingo, we endeavored to unveil the role of AI-based language learning in elevating speaking skills and fostering self-regulation among Chinese EFL learners.

### 3.3. Procedure

In this study, we employed a comparative design to investigate the impact of an AI-based language learning application, Duolingo, on speaking skills and self-regulation in the Chinese EFL context. The participants were randomly assigned to either the intervention group or the control group.

The intervention group received instruction using the Duolingo application, which utilizes natural language processing technology to facilitate language learning across multiple skills, including speaking, listening, reading, and writing. Specifically, our study focused on the speaking component of the application. The Duolingo AI chatbot played a central role in this intervention by providing learners with prompts and questions in English. The learners were then required to respond to these prompts, and the chatbot provided real-time feedback on various aspects of their spoken language, including pronunciation, fluency, vocabulary, and accuracy. This feedback was generated through machine learning algorithms that analyzed learners’ performance data, enabling the chatbot to offer personalized feedback tailored to each learner’s specific needs. The intervention also included group activities and discussions, allowing learners to practice their speaking skills in a more naturalistic setting. Furthermore, learners had the opportunity to track their progress within the Duolingo application, which provided them with motivation and feedback on their overall language learning journey.

In contrast, the control group received instruction through a more traditional speaking course. This course focused on facilitating speaking skills development through group discussions, activities, role plays, and presentations. Although this course provided learners with opportunities to practice their speaking skills in a supportive and structured environment, it did not utilize AI technology or offer personalized feedback on learners’ speaking performance.

To ensure the integrity and validity of our study, both the intervention and control groups received an equal amount of instructional time and were exposed to comparable learning contexts, except for the differing instructional methods described above. By comparing the outcomes of the intervention and control groups, we aimed to examine the specific impact of AI-powered language learning on speaking skills and self-regulation in the Chinese EFL context.

### 3.4. Treatment adherence

In this study, the consistent and accurate delivery of the AI-based speaking intervention was paramount. To ensure treatment adherence and monitor treatment fidelity across all participating institutes and groups, rigorous procedures were implemented (Moncher and Prinz, 1991).

Initially, comprehensive training sessions were conducted for both the researchers and the English teachers responsible for delivering the AI-based speaking instruction. This training encompassed detailed instructions on how to effectively utilize the Duolingo application, facilitate the speaking activities, and provide constructive feedback to learners. Subsequently, an initial evaluation was conducted to assess the teachers’ preparedness, knowledge of course materials, and proficiency in using the AI-based platform.

Continuous monitoring of treatment fidelity was achieved through a series of unannounced observations during the intervention period. These observations were conducted randomly at various points throughout the study at each participating institute. The primary objective was to verify that the speaking activities were being delivered in accordance with the intended design. To further ensure consistency in delivery, a checklist (see the Appendix) was devised to monitor the execution of speaking instruction in both the intervention and control groups. This checklist covered essential aspects such as the duration of the speaking activities, the specific types of activities employed, and the nature of feedback provided to learners. English teachers responsible for delivering the speaking instruction diligently completed this checklist for each session.

The proficiency of the English teachers in delivering the intervention and control courses was regularly evaluated by the research team. This evaluation included assessing the teachers’ knowledge of course materials, their facilitation of group discussions, and their ability to provide effective feedback to learners. These evaluations were carried out periodically to ensure the ongoing quality of instruction. Throughout the study, strict adherence to the intended intervention protocol was maintained. Any deviations from the prescribed intervention were meticulously documented and addressed. The rigorous monitoring of treatment fidelity was essential to uphold the validity and reliability of the study’s findings, ensuring that any observed differences in outcomes between the intervention and control groups could be confidently attributed to the intervention itself rather than variations in instructional delivery.

Additionally, in order to maintain experimental fidelity and prevent potential contamination of the control group, experimental students were placed in separate classrooms from the control group. This physical separation ensured that experimental students did not share access or inadvertently influence control group participants. Additionally, students were reminded of the importance of maintaining confidentiality and not discussing specific instructional details or materials outside of their respective groups. The research team also conducted periodic checks to monitor and reinforce compliance with these guidelines throughout the study duration.

### 3.5. Data analysis

The study used several analyzes to examine the effectiveness of the intervention and ensure the groups were similar at the beginning of the study. First, two-tailed *t*-tests were conducted for all dependent and control variables to examine baseline equivalence. The purpose was to ensure that any differences observed between the groups at the end of the study were due to the intervention and not pre-existing differences.

To evaluate the usefulness of the intervention, multiple linear regressions were used. Mplus Version 7 was used to conduct the analyzes employing maximum likelihood robust estimation (MLR). The amount of missing data varied between 1.8 and 5.7%, with the higher rate resulting from students’ absence at posttest. However, there was no differential dropout between the treatment and control group (*χ*^2^(1, 93) = 1.08; *p* = 0.263), which suggests that the missing data were missing at random. To handle missing data, the full-information maximum likelihood (FIML) estimator was employed.

For evaluating the effects of the training, directed hypotheses were formulated, and one-tailed tests of significance were employed. The significance level (α) was set at 0.05 to determine the statistical significance of the findings. This approach was used to examine whether the intervention had a positive effect on the speaking skill components based on IELTS: fluency, vocabulary, accuracy, and pronunciation. Also, the effect of AI-based intervention on self-regulation was investigated. In order to enhance the accuracy of the regression coefficients and mitigate any potential bias stemming from initial between-group differences, control variables were incorporated. These covariates encompassed overall English proficiency, speaking anxiety, and pretest performance.

The dependent variables were measured as posttest measurements for each of the four variables of speaking skills. To assess the effects of pretest differences and differential effects for participants with low versus high pretest scores on the dependent variable, the pretest score and interaction term of course and pretest score were included as additional predictor variables. If there was a significant interaction term, the effect of course participation differed for students depending on their initial score on the dependent variable. In order to evaluate the impact of initial score discrepancies and divergent outcomes among participants with varying pretest scores, additional predictor variables were incorporated. These variables consisted of the pretest score itself and the interaction term between the course and pretest score. In the event that a substantial interaction term was identified, the influence of course participation varied for students based on their initial performance on the dependent variable (Cohen et al., 2003). All continuous variables were standardized before analysis. Each course was binary coded, with speech training coded as 1 and the control group as 0. The size of the course or treatment effect was indicated by the standardized mean differences between the two groups, calculated using Hedges (2007).

## 4. Results

Table 1 shows the means and standard deviations for each group in the pre-and post-tests. The table presents the data for the dependent variables, which are the four speaking skills (i.e., fluency, vocabulary, accuracy, and pronunciation) and one control variable (i.e., speaking anxiety). Additionally, the CET scores are reported, which were taken as a measure of English language proficiency.

**Table 1:** Means and standard deviations for each group in pre- and post-tests.

	Pre-test				Post-test			
	Experimental G		Control G		Experimental G			Control G
	M	SD	M	SD	M	SD	M	SD
Fluency	5.12	0.62	5.24	0.59	5.94	0.49	5.52	0.67
Vocabulary	4.85	0.78	4.73	0.69	5.78	0.67	5.09	0.63
Accuracy	5.26	0.81	5.32	0.58	6.32	0.93	4.97	0.56
Pronunciation	4.77	0.64	4.63	0.73	5.91	0.77	5.13	0.92
Self-regulation	3.28	0.73	3.41	0.71	4.34	0.82	3.89	0.83
CET	42.35	11.26	43.58	12.06				
Speaking anxiety	3.12	0.69	2.94	0.57				

Upon close examination of the pre-test means presented in Table 1, it is observed that there were slight group differences across some variables. However, to determine the statistical significance of these variations, independent samples t-tests were conducted. The results of these tests indicated that there were no statistically significant differences between the experimental and control groups on any of the pre-test variables (*p* > 0.05). This rigorous analysis confirms the initial equivalence of the groups at the outset of the study, ensuring that any subsequent changes in speaking skills and self-regulation can be attributed to the intervention rather than pre-existing disparities in proficiency levels. The table also shows that, at pretest, the means for each group on all variables were relatively similar, with no significant differences between the groups. This suggests that the groups were equivalent at the beginning of the study. After the intervention, the experimental group showed higher means than the control group on all speaking skills as well as self-regulation, suggesting a positive effect of the intervention on these skills. However, the two groups did not differ significantly on the control variable of speaking anxiety.

Table 2 presents the correlations between variables at the pretest and posttest for the study. The results indicate that at the pretest, fluency is significantly correlated with any other variables. Also, at the posttest, fluency is significantly correlated with vocabulary (*r* = 0.36, *p* < 0.01), accuracy (*r* = 0.24, *p* < 0.05), and pronunciation (*r* = 0.33, *p* < 0.01). In addition, at the posttest, vocabulary is significantly correlated with fluency (*r* = 0.28, *p* < 0.05), accuracy (*r* = 0.34, *p* < 0.01), and pronunciation (*r* = 0.22, p < 0.05). Accuracy is significantly correlated with fluency (*r* = 0.27, *p* < 0.05) and vocabulary (*r* = 0.22, *p* < 0.05) at the pretest, and with all other variables at the posttest (*r* = 0.41 to 0.35, *p* < 0.01). Also, pronunciation is also significantly correlated with all other variables at the posttest (*r* = 0.37 to 0.35, *p* < 0.01). Likewise, self-regulation was positively correlated with all the other constructs both at pre-and posttest.

**Table 2:** Correlations between the variables at the pretest (below diagonal) and the posttest (above diagonal).

	1	2	3	4	5
(1) Fluency	–	0.36**	0.24*	0.33**	0.34**
(2) Vocabulary	0.31**	–	0.28*	0.34**	0.22**
(3) Accuracy	0.27*	0.22*	–	0.41**	0.32**
(4) Pronunciation	0.37**	0.21*	0.35**	–	0.38**
(5) Self-regulation	0.32**	0.27**	0.36**	0.43**	–

*p < 0.05; **p < 0.01.

Table 3 reports the results of examining the effects of an AI-based instruction on L2 speaking skills. The results indicate that the course had a significant positive effect on three of the four dependent variables: fluency (*B* = 0.65, SE = 0.24, *p* = 0.006), vocabulary (*B* = 0.57, SE = 0.29, *p* = 0.034), and accuracy (*B* = 0.46, SE = 0.23, *p* = 0.013). Also, the course had a significant effect on pronunciation (*B* = 0.42, SE = 0.21, *p* = 0.025). In addition, the intervention was found to have a significant effect on self-regulation (*B* = 0.51, SE = 0.22, *p* = 0.018). This suggests that controlling for the pretest scores and the interaction term between the course and pretest score, global English proficiency, and speaking anxiety, students who engaged in the speech training demonstrated significantly elevated scores in both speaking skills and self-regulation in comparison to students in the control group.

**Table 3:** AI-based instruction effects on the L2 speaking skills (posttest).

	Fluency			Vocabulary			Accuracy			Pronunciation			Self-regulation		
	*B*	(SE)	*p*	*B*	(SE)	*p*	B	(SE)	*p*	*B*	(SE)	*p*	*B*	(SE)	*p*
Course	0.65	0.24	0.006	0.57	0.29	0.034	0.46	0.23	0.013	0.42	0.21	0.025	0.51	0.22	0.018
Pretest Score	0.39	0.21	0.112	0.19	0.22	0.427	0.42	0.17	0.042	0.53	0.21	0.016	0.46	0.23	0.027
Course × Pretest Score	−0.31	0.22	0.346	0.41	0.29	0.046	0.11	0.14	0.573	−0.26	0.15	0.456	0.13	0.15	0.462
CET	0.28	0.16	0.308	0.19	0.11	0.296	−0.24	0.16	0.954	0.15	0.11	0.372	0.14	0.16	0.764
Speaking anxiety	−0.34	0.16	0.036	0.12	0.13	0.826	−0.33	0.17	0.307	−0.28	0.16	0.046	−0.16	0.15	0.359
Explained variance (*R*^2^)	0.44			0.33			0.26			0.23			0.28		

The pretest score had a significant positive effect on accuracy (*B* = 0.42, SE = 0.17, *p* = 0.042), pronunciation (*B* = 0.53, SE = 0.21, *p* = 0.016), and self-regulation (*B* = 0.46, SE = 0.23, *p* = 0.027), but not on fluency or vocabulary. Concerning the varying effects based on students’ initial assessment scores, a statistically significant interaction term between the pretest score and course was observed solely for the vocabulary component (*B* = 0.41, *p* = 0.046). Accordingly, students with higher vocabulary level at the pretest benefitted more from the course.

Among the control variables, speaking anxiety had a significant negative effect on fluency (*B* = −0.34, SE = 0.16, *p* = 0.036) and pronunciation (*B* = −0.28, SE = 0.16, *p* = 0.046). This suggests that higher levels of speaking anxiety were associated with decreased fluency and less accurate pronunciation during the speaking tasks. This finding underscores the importance of addressing and mitigating speaking anxiety in language learning contexts to enhance learners’ oral proficiency. In contrast, speaking anxiety did not demonstrate a significant effect on vocabulary, accuracy, or self-regulation. This implies that while speaking anxiety may exert a notable influence on certain facets of speaking skills, it may not necessarily impact other linguistic domains or learners’ self-regulation strategies. These results suggest that learners’ ability to select and deploy vocabulary, maintain grammatical accuracy, and engage in self-regulated learning processes may be less directly influenced by their level of speaking anxiety.

Also, CET did not have a significant effect on any of the dependent variables. The explained variance (*R*^2^) for each dependent variable was moderate, ranging from 0.23 for pronunciation to 0.44 for fluency, indicating that the independent variables accounted for a significant portion of the variance in the dependent variables.

## 5. Discussion

This research employed a mixed-methods approach to gather and analyze data, drawing upon Vygotsky’s (1984) social constructivism to investigate the effects of AI-supported instruction on EFL learners’ speaking skills and self-regulation in speaking. The quantitative findings initially demonstrated that AI-based instruction had a significant positive impact on learners’ speaking skills, surpassing its non-AI counterpart. These results align with prior studies by Hill et al. (2015), Junaidi (2020), Kang (2022), and Suciati et al. (2022), which also reported favorable effects of AI on students’ speaking abilities. It is plausible that learners’ engagement with AI in more stimulating and interactive ways played a role in enhancing their speaking skills. In other words, the virtual experience with AI motivated the learners to engage in communication in a novel environment, potentially contributing to the observed improvements in their speaking proficiency.

AI-based instruction offers learners personalized and adaptive learning experiences, facilitating the analysis of their performance and enabling the identification of areas for improvement, as well as the provision of tailored feedback and practice materials (Dupoux, 2018; Yan, 2023). This individualized approach allows learners to address their specific language needs and progress at their preferred pace, thereby fostering enhanced language development (Yang et al., 2021). Furthermore, AI-based instruction provides learners with extensive and immersive language input through various means such as interactive simulations, virtual environments, and AI-powered chatbots or language tutors (Jia et al., 2022). Engaging in realistic and contextually rich speaking activities within these platforms exposes learners to authentic language use, which plays a pivotal role in facilitating the development of fluency, vocabulary, and pragmatic skills necessary for effective oral communication (Bahrani and Sim, 2012). In addition, AI-based instruction ensures learners receive continuous and immediate feedback on their speaking performance (Dodigovic, 2007; Kim et al., 2019). By leveraging AI technologies, such as advanced speech recognition and language processing algorithms, learners’ pronunciation, grammar, and discourse features can be analyzed, enabling the provision of real-time corrective feedback (Dodigovic, 2005; Divekar et al., 2022). This prompt feedback allows learners to promptly identify and rectify errors, reinforcing accurate language production and fostering the development of self-monitoring and self-correction skills (Li, 2023; Loncar et al., 2023). The findings of this study demonstrate that AI-supported interactions fostered the development of EFL learners’ self-regulation and outperformed learner-learner interactions in the control group. These findings align with Vygotsky’s (1984) social constructivism, highlighting the role of AI as a facilitator in the growth of students’ self-regulation. Consistent with Vygotsky’s recommendations, learners initially engaged in communicative speaking activities with AI, which likely assisted them in regulating their own speaking performance. Through these communicative activities, students gradually transitioned from other-regulation to self-regulation, demonstrating independent speaking performance. Notably, the students who exhibited self-control were able to complete their speaking tasks without relying on AI or other students, indicating higher levels of self-regulation among the AI learners. More specifically, in relation to Vygotsky’s sociocultural theory (1986), the idea of scaffolding emphasizes the importance of external support in aiding learners’ cognitive and linguistic growth. AI systems can serve as valuable scaffolds by offering learners customized prompts, reminders, and feedback that address their specific needs. This personalized assistance enables learners to regulate their learning, establish goals, track their progress, and adapt accordingly, ultimately facilitating the development of self-regulation skills.

Furthermore, according to Bandura’s (1989) social cognitive theory, learning occurs through the observation, imitation, and modeling of others’ behaviors. In the context of interactions supported by AI, learners have the opportunity to observe and engage with AI systems that demonstrate self-regulatory behaviors, such as offering adaptive feedback or guiding learners in goal-setting and planning. By observing these behaviors, learners can internalize and replicate self-regulatory strategies, leading to the development of their own self-regulation skills. Moreover, AI technologies provide certain advantages, such as adaptive learning algorithms and real-time data analysis, which enable learners to receive immediate feedback and monitor their performance. This prompt feedback allows learners to evaluate their progress, identify areas in need of improvement, and adapt their learning strategies accordingly. By engaging in self-reflection and making adjustments based on the feedback received from AI systems, learners can cultivate metacognitive awareness and self-regulatory behaviors (Zimmerman, 2002).

In examining the effects of AI-based instruction on self-regulation, it is imperative to consider the stability of self-regulation over time. Self-regulation is a dynamic construct that may evolve and fluctuate based on various factors, including the duration and intensity of the intervention (Zimmerman and Moylan, 2009). Research suggests that achieving meaningful improvements in self-regulation capability often requires a sustained effort and a considerable investment of time (Metcalfe and Mischel, 1999; Zimmerman, 2013; Duckworth et al., 2019).

While our study demonstrates significant positive effects of AI-based instruction on self-regulation among Chinese EFL learners, it is important to acknowledge that the duration and intensity of the intervention may influence the stability of these gains. Previous studies have indicated that extended exposure to self-regulation interventions, along with consistent practice and reinforcement, is crucial for enduring improvements in self-regulatory skills (Zimmerman and Schunk, 2011; Duckworth et al., 2019; Lei et al., 2022). Moreover, individual differences in learners’ receptiveness to self-regulation strategies and their capacity for sustained effort may play a role in the duration required to observe significant changes. Factors such as prior experience with self-regulation techniques, intrinsic motivation, and external support systems can influence the rate at which individuals develop and consolidate self-regulation skills (Zimmerman, 2002).

The observed improvements in students’ speaking skills and self-regulation can be attributed to the flexibility of engaging in communicative speaking activities with AI anytime and anywhere. Unlike traditional classroom settings, learners were not limited by time and location, allowing them to engage in interactive speaking activities at their convenience. These findings align with Gardner et al. (2021) and Hamuddin (2018), who affirmed the positive role of AI in providing learners with opportunities to communicate in various convenient settings and at flexible times. Furthermore, students were more inclined to communicate with AI due to the stress-free environment it provided for collaborative speaking activities. Speaking anxiety often hinders learners from actively participating in interactive speaking activities with instructors and peers. In this context, AI facilitated greater engagement in communicative speaking activities, which, in turn, contributed to the development of students’ speaking skills and self-regulation.

These findings align with the research conducted by Kang (2022), which also confirmed learners’ favorable effects of AI-supported instruction. EFL students might have found interacting with AI to be more enjoyable compared to communicating with their peers, which likely contributed to their enhanced performance in speaking activities. As highlighted by Sun et al. (2021) and Zhang (2022), the AI environment offered learners various user-friendly features, including portability and accessibility at any time and place. These features may have contributed to the AI learners’ greater improvements in speaking skills and self-regulation compared to non-AI learners.

The implementation of an AI environment is recommended due to its potential to create a motivating and engaging technological setting, enabling students to interact more effectively with AI and their peers, thereby enhancing their speaking skills and self-regulation. The findings of this study indicate that EFL students actively participated in collaborative speaking activities with AI and their peers, leading to significant improvements in their speaking skills and self-regulation. The interactive speaking tasks facilitated by AI likely played a role in enhancing students’ ability to self-regulate their speaking skills. According to Vygotsky (1984), students with diverse abilities can assist their peers in reaching higher levels of learning performance, including self-regulation. In the context of this study, AI also supported students in successfully performing interactive speaking tasks by fostering positive evaluations of their speaking abilities, aligning with the findings of Suciati et al. (2022).

Finally, the findings regarding the impact of speaking anxiety on different aspects of speaking skills provide valuable insights into the complexity of this phenomenon. Specifically, higher levels of speaking anxiety were found to have a significant detrimental effect on fluency and pronunciation, indicating that learners experiencing greater anxiety tend to exhibit reduced fluency and less accurate pronunciation during speaking tasks. This underscores the critical need to address and alleviate speaking anxiety in language learning environments, as it directly correlates with learners’ oral proficiency (Galante, 2018).

However, it is noteworthy that speaking anxiety did not yield a significant effect on vocabulary, accuracy, or self-regulation. This suggests an intricate relationship between speaking anxiety and various linguistic and self-regulation domains. While anxiety may exert a notable influence on the fluidity and pronunciation of speech, it may not necessarily impede other facets of language acquisition, such as lexical selection, grammatical accuracy, or self-regulated learning processes (Pae, 2013). These results indicate that learners may possess a degree of resilience in certain linguistic areas, demonstrating an ability to select and utilize vocabulary, maintain grammatical precision, and engage in self-regulated learning practices, irrespective of their level of speaking anxiety. This nuanced understanding of the relationship between anxiety and different aspects of speaking skills can inform targeted interventions to enhance overall oral proficiency and mitigate the adverse effects of speaking anxiety on language learners.

## 6. Conclusion and implications

The primary aim of the present study was to investigate the potential of AI in enhancing EFL students’ speaking skills and self-regulation. The findings indicated that AI learners exhibited greater improvements in both speaking skills and self-regulation compared to non-AI learners. These positive outcomes can be attributed to the creative and engaging environment that AI provided for interactive speaking activities. The use of the Duolingo application, which incorporates AI technologies such as natural language processing, interactive exercises, personalized feedback, and speech recognition, resulted in significantly greater improvement in L2 speaking skills than traditional instruction. This suggests that AI-based instruction has the potential to enhance language learning by providing learners with interactive and personalized learning experiences that target specific language areas for improvement. Also, the participants in the experimental group, who received AI-based instruction, reported higher levels of self-regulation than the control group. This indicates that AI technologies can support learners in regulating their learning processes, setting goals, monitoring their progress, and making necessary adjustments. Having offered personalized feedback and adaptive exercises, AI-based instruction empowers learners to take control of their learning and develop metacognitive strategies that enhance their speaking skills.

The results of this study have important implications for EFL education. Given that AI aligns with student-centered approaches and significantly enhances EFL students’ speaking skills and self-regulation, its integration is recommended in interactive EFL speaking courses. EFL educators are encouraged to incorporate AI in their communicative speaking courses to facilitate the development of speaking skills and self-regulation among EFL students. By implementing an AI-supported classroom, EFL teachers can design engaging communicative speaking activities and tasks involving both AI and peers. This approach would enable EFL students to engage in meaningful speaking interactions, thereby improving their speaking skills and self-regulation.

EFL students can benefit greatly from an AI-infused course specifically designed for communicative speaking activities. By participating in such a course, students can receive feedback from both peers and AI, leading to improvements in their communication abilities and self-regulation in speaking. Additionally, the engaging nature of the AI environment’s communicative speaking activities is likely to enhance EFL students’ speaking proficiency and foster their enthusiasm for further language learning endeavors. The findings suggest that AI-based instruction holds promise for addressing the unique challenges faced by Chinese EFL learners in developing speaking skills and self-regulation. With the growing demand for English proficiency in China, integrating AI technologies into language classrooms can provide learners with effective tools to improve their speaking abilities and develop important self-regulatory competencies.

Despite its implications, this study has several limitations that should be considered. Firstly, the generalizability of the findings may be restricted to the specific sample of Chinese EFL students in a natural setting, cautioning against applying these results to learners from different language backgrounds or diverse cultural and educational contexts. Secondly, the relatively small sample size of 93 participants might have compromised the statistical power and confidence in the findings, highlighting the need for larger samples to enhance generalizability and detect smaller effects more reliably.

Another limitation pertains to the duration of the AI-based instruction and the length of exposure to the intervention, as the impact on speaking skills and self-regulation could vary depending on the intervention’s duration. Longer intervention periods may yield different outcomes and provide a more comprehensive understanding of the effects. Additionally, the control group in this study received traditional instruction, which introduces the possibility of confounding factors influencing the observed differences between the experimental and control groups. To better isolate the effects of AI-based instruction, future research could incorporate an active control group that receives an alternative instructional approach.

While the study employed pre-and post-tests to assess L2 speaking skills and self-regulation abilities, it is important to acknowledge that these measures may not fully capture the complexity and range of these skills. Including multiple measures and qualitative assessments in future studies would provide a more comprehensive understanding of the impact of AI-based instruction. The study also recognizes the need for further research to investigate the long-term effects of AI-based instruction and delve into the specific mechanisms underlying the observed improvements. Gaining insight into the sustainability of the effects and understanding the underlying processes will contribute to a more robust body of evidence regarding the effectiveness of AI-based instruction in language learning.

Although our study primarily focused on the positive effects of AI-based instruction on speaking skills and self-regulation, we recognize the need for continued attention to the complex issue of speaking anxiety in language learning. It is important to acknowledge that while our study did not find a significant change in speaking anxiety as a result of the AI-based intervention, the negative impact of speaking anxiety on fluency and pronunciation highlights the importance of addressing learners’ anxiety levels in language learning contexts. Understanding its impact and exploring effective strategies to alleviate it can further enhance the language learning experience for students. Additionally, the potential differences in anxiety levels between practicing with an AI tool and a human instructor in real conversations present an intriguing avenue for further research. Future studies could explore the comparative effectiveness of practicing with AI tools and human instructors in reducing anxiety and enhancing conversational proficiency. This research could shed light on the unique benefits and challenges associated with each approach, ultimately providing valuable insights for language learners and educators.

## Data availability statement

The raw data supporting the conclusions of this article will be made available by the authors, without undue reservation. Requests to access these datasets should be directed to AZ, 60289@bttc.edu.cn.

## Ethics statement

The studies involving humans were approved by Department of Foreign language, Baotou Teachers’ College, Inner Mongolia University of Science and Technology, China. The studies were conducted in accordance with the local legislation and institutional requirements. The participants provided their written informed consent to participate in this study. Written informed consent was obtained from the individual(s) for the publication of any potentially identifiable images or data included in this article.

## Author contributions

HQ: Data curation, Investigation, Methodology, Resources, Software, Validation, Writing – original draft, Writing – review & editing. AZ: Conceptualization, Formal analysis, Project administration, Supervision, Visualization, Writing – original draft.

## Appendix

Treatment adherence observation checklist

*Instructions for observers*:

The following checklist was used to monitor the consistent and accurate delivery of the AI-based speaking intervention. Observers were instructed to conduct unannounced observations at various points throughout the study to verify that the speaking activities were being delivered in accordance with the intended design.

*Checklist items*:

Duration of speaking activity:

Met the prescribed time frame.

Deviated from the prescribed time frame (provide details):

Types of speaking activities:

Implemented the specified activities as outlined in the intervention protocol.

Deviated from the specified activities (provide details):

Nature of Feedback Provided to Learners:

Provided constructive and relevant feedback to learners.

Did not provide adequate feedback (provide details):

*Additional comments*:
